# Tubuloglomerular Disease With Cone-Shaped Epiphyses Associated With Hypomorphic Variant and a Novel p.Cys14Arg in the *TTC21B* Gene: A Case Report

**DOI:** 10.3389/fped.2021.752878

**Published:** 2021-11-05

**Authors:** Martin Bezdíčka, Dana Zemková, Sylva Skálová, Eva Hovorková, Miroslav Podhola, Jan Burkert, Jakub Zieg

**Affiliations:** ^1^Vera Vavrova Lab/VIAL, Department of Pediatrics, Second Faculty of Medicine, Charles University and Motol University Hospital, Prague, Czechia; ^2^Department of Pediatrics, Second Faculty of Medicine, Charles University and Motol University Hospital, Prague, Czechia; ^3^Department of Pediatrics, Faculty of Medicine in Hradec Králové, Charles University and Hospital Hradec Králové, Hradec Králové, Czechia; ^4^Department of Pathology, Faculty of Medicine in Hradec Králové, Charles University and Hospital Hradec Králové, Hradec Králové, Czechia; ^5^Department of Cardiovascular Surgery, Second Faculty of Medicine, Charles University and Motol University Hospital, Prague, Czechia

**Keywords:** cone-shaped epiphyses, nephrotic syndrome, *TTC21B*, podocyte, proteinuria, case report

## Abstract

Monogenic nephrotic syndrome (NS) is associated with a resistance to initial glucocorticoid therapy and causative variants, which may be found in several genes influencing podocyte stability and kidney development. The *TTC21B* gene, which encodes the retrograde intraflagellar transport protein IFT139, is found mostly in association with ciliopathies in humans. The role of this protein in podocyte cytoskeleton stability was confirmed later and the mutated *TTC21B* also may be associated with proteinuric diseases, such as nephrotic syndrome. Our patient manifested as an infant with brachydactyly, nephrotic-range proteinuria, and renal tubular acidosis, and a kidney biopsy revealed focal segmental glomerulosclerosis (FSGS). Multiple phalangeal cone-shaped epiphyses of the hand were seen on X-ray. Next-generation sequencing revealed the well-described p.Pro209Leu heterozygous variant and a novel heterozygous p.Cys14Arg variant in the *TTC21B* gene. Our finding confirmed that the causative variants in the *TTC21B* gene may contribute to a spectrum of clinical features, such as glomerular proteinuric disease with tubulointerstitial involvement and skeletal abnormalities.

## Introduction

Nephrotic syndrome (NS) is characterized by massive proteinuria, hypoalbuminemia, edema, and dyslipidemia (1). Nephrotic syndrome is one of the most common rare pediatric kidney diseases, with an estimated incidence of 1:25,000 children annually (2). There are two main subtypes of NS, divided according to the response to an initial 6 weeks of glucocorticoid therapy (prednisone 60 mg/m^2^/day): steroid-sensitive NS and steroid-resistant NS (SRNS) (2). SRNS comprises 20% of all NS cases that do not reach remission and are at high risk for end-stage kidney disease (ESKD) (3). Moreover, almost one-third of SRNS patients carry a pathogenic variant in one of the approximately 70 genes associated with the disease (4, 5). Among the most mutated genes (*NPHS1, NPHS2, WT1*), there are many other less common, such as the *TTC21B* gene.

The *TTC21B* gene encodes the retrograde intraflagellar transport protein IFT139, which is important in cooperation with dynein 2 for the proper formation of primary cilium (6). The primary cilium is an immotile microtubular structure that occurs on the apical membranes of most cells and has many critical roles, such as sensing physical or biochemical extracellular signals for the cell (6). There are functional studies that have verified that mutated IFT139 is associated with the shortening of cilia, a mechanism that disrupts the function of this organelle (7). Therefore, pathogenic variants in *TTC21B* are mainly associated with ciliopathies, such as nephronophthisis and asphyxiating thoracic dystrophy (Jeune syndrome), characterized by skeletal abnormalities and various organ involvement (7). Further investigation expanded this association to a specific case of familiar focal segmental glomerulosclerosis (FSGS) associated with tubulointerstitial lesions and sometimes, other extrarenal features, such as myopia (8–10).

Here, we present a patient with SRNS with a unique phenotype carrying a known *TTC21B* p.Pro209Leu hypomorphic variant in trans with a novel p.Cys14Arg variant.

## Case Report

**Figure 1:**
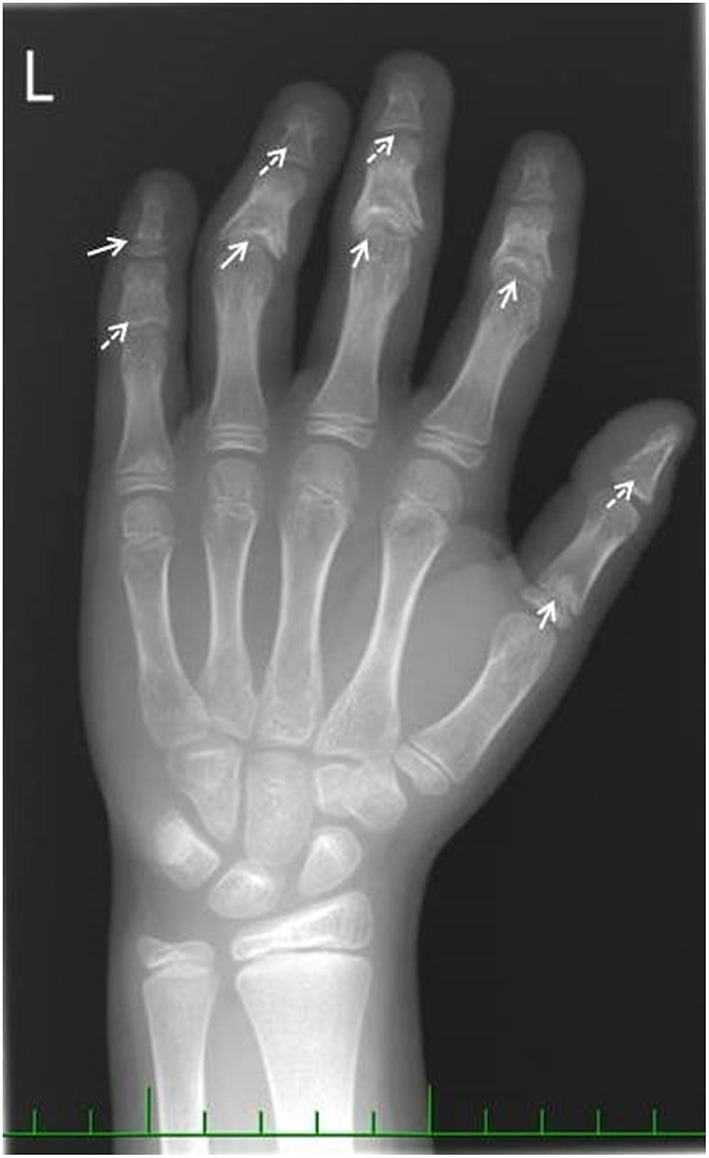
Hand X-ray image. Full arrows show coned epiphyses. Dashed arrows show the premature closure growth plates due to coned epiphyses.

A 2.5-year-old girl with nephrotic-range proteinuria and progressive deterioration of renal function was referred to our tertiary pediatric nephrology center. Her family history was unremarkable. She presented after birth with transient renal tubular acidosis and mild proteinuria (mostly glomerular), which, at the age of 6 months, reached nephrotic range. Her kidney function and blood pressure were normal. Initial ultrasound showed kidneys of appropriate size with normal parenchymal echogenicity. She was started on the ACE inhibitor ramipril, and oral alkali therapy was administered for 3 months. On admission, she was hypertensive (121/80), her height and weight were both in the 50th percentile for age, and her physical examination was normal, apart from specific skeletal abnormalities (brachydactyly and disproportional growth with longer trunk and shorter extremities). The X-ray showed coned epiphyses of the fingers of her hand (Figure 1). Laboratory investigation revealed normal serum albumin (37.5 g/l), elevated markers of kidney function (urea, 14.2 mmol/l; creatinine, 155 μmol/l), and normal iontogram and acidobasic balance parameters (sodium, 140 mmol/l; potassium, 5.2 mmol/l; calcium, 2.27 mmol/l; magnesium, 0.92 mmol/l; pH, 7.384; bicarbonate, 19 mmol/l). Urinalysis revealed no hematuria, normal calcium/creatinine ratio of 0.18 mmol/mmol, and elevated total protein/creatinine ratio of 463 mg/mmol. Molecular genetic analysis [Sanger sequencing of *NPHS2* and *WT1* (exon 8 and 9) genes] did not find any causative variants. The percutaneous kidney biopsy finding was consistent with the histological features of FSGS. Fifty percent of the glomeruli was globally sclerosed, and the remaining glomeruli exhibited mesangial expansion, segmental glomerulosclerosis, and periglomerular concentric fibrosis. Of the interstitium, 70% was affected by fibrosis and tubular atrophy. Weak positivity of IgM was visible by immunofluorescence at the sclerotic glomeruli. Electron microscopy revealed diffuse pedicular fusion. Conservative therapy of chronic kidney disease (darbepoetin, calcitriol, amlodipine) was introduced. Chronic peritoneal dialysis was started after two months due to significant deterioration of kidney function. Our patient underwent successful cadaveric kidney transplantation 9 months later. Her post-transplant course was uneventful until the last observation (at age 6 years). Due to the unknown etiology of the primary nephropathy in our child, further genetic testing was indicated.

**Figure 2:**
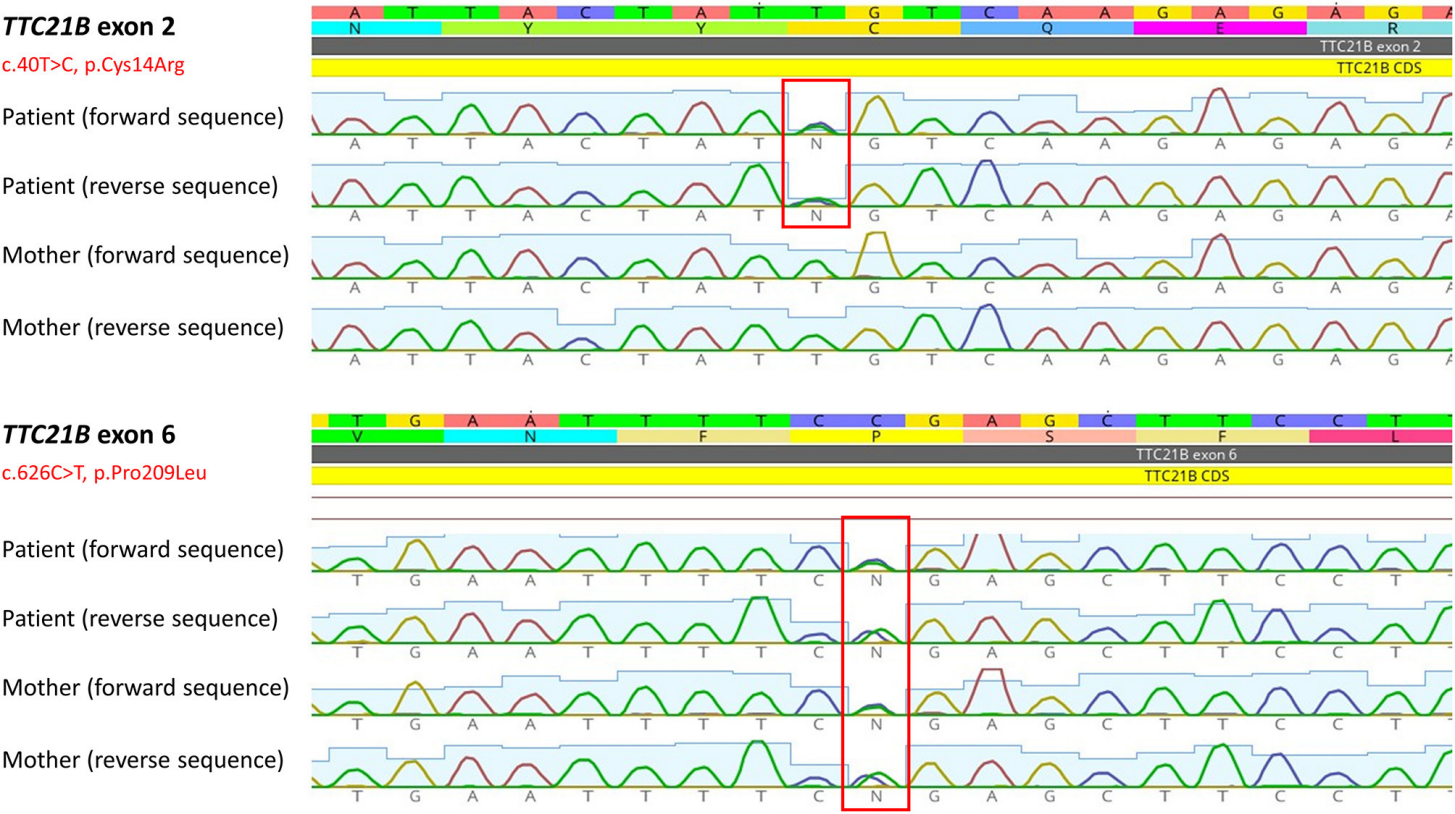
Two heterozygous *TTC21B* variants found in the patient. Sequencing of maternal sample revealed p.Pro209Leu heterozygous variant, but not the novel p.Cys14Arg variant. The image was obtained by Geneious Prime software (Biomatters, Ltd., New Zealand).

**Figure 3:**
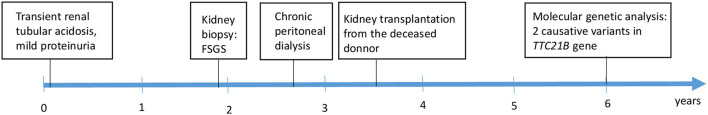
Clinical course of the patient.

The DNA sample of the patient was analyzed by next generation sequencing. The libraries were prepared according to the manufacturer's protocol and sequenced with a NextSeq 500/550 High Output Kit v2.5 (150 cycles) on a NextSeq 550 instrument (Illumina, San Diego, CA). The sequenced data were filtered and evaluated using the Varaft annotation and filter tool according to the current standards of the American College of Medical Genetics and Genomics, as previously described (11). The analysis revealed a known heterozygous variant c.626C>T, p.Pro209Leu (rs140511594) in trans with a novel variant c.40T>C, p.Cys14Arg (rs780354129; very rare variant according to frequency databases) in *TTC21B* gene (NM_024753.5, NP_079029.3) (Figure 2). The evidence of pathogenicity by prediction programs was determined: UMD predictor = Pathogenic, Sift = Damaging, Polyphen-2 = Probably Damaging, LRT = Deleterious, Mutation Taster = Disease Causing, Provean = Damaging, M-Cap = Damaging, CADD score = 26.9 (variants with a score over 20 are usually pathogenic). No other causative variants in known SRNS or FSGS genes were found. Sanger sequencing of the maternal DNA sample confirmed the presumed inheritance of the variant. The patient's mother is a healthy carrier of the c.626C>T, p.Pro209Leu heterozygous variant. Unfortunately, the DNA of the father was unavailable, but since the second *TTC21B* variant was not found in the maternal sample, we could predict the autosomal recessive manner of the inheritance. A timeline illustrates the course of the disease for our patient (Figure 3).

## Discussion

Patients with monogenic NS may present with isolated forms (e.g., mutated *NPHS1, NPHS2*), but there are also many syndromic forms of NS caused by different mutated genes (4). Causal variants in the *WT1* gene are responsible for the most known syndromic NS cases (e.g., Denys-Drash or Frasier syndrome), characterized by disorders of sex development and the risk of developing nephroblastoma (12). Other syndromic NS are connected to pathogenic variants in other genes: *E2F3* (NS with intellectual disability), *LMX1B* (nail patella syndrome), *SMARCAL1* (NS with bone dysplasia and immunodeficiency), or *WDR73* (NS with microcephaly) (4). Our patient did not manifest with all the expected features of NS, which was not unusual because patients with genetic proteinuric nephropathies may often present without edema (13). Nephrotic-range proteinuria and FSGS on kidney biopsy, a common histological finding in patients with SRNS (4, 5), led us to analyze the genes associated with NS. The revelation of the causative variants in *TTC21B* clarified the cause of NS with extrarenal features in our patient. Mouse embryo *TTC21B* null mutant showed sonic hedgehog pathway dysregulation and a phenotype resembling some human ciliopathies (14). Another *in vivo* study described zebrafish embryos with blocked translation of *TTC21B* and several defects (shortening of the embryonic axis, widening and kinking of the notochord, and broadening and thinning of the somites) (7). The phenotype of the zebrafish embryos was rescued by the injection of human *TTC21B* mRNA, but only a partial rescuing by *TTC21B* mRNA containing the p.Pro209Leu homozygous variant was achieved. A similar result was attained in a rescue test of murine inner medullary-collecting duct cells with the IFT139 expression blocked by shRNA, which led to the reduction of cell cilia. The rescue test by plasmid containing p.Pro209Leu variant showed partial rescue of the cilia (7). These observations confirm the pathogenic role of the p.Pro209Leu variant in cilium stability and also explain the cause of ciliopathies, such as in patients with nephronophthisis or Jeune syndrome.

The role of podocyte damage in patients with *TTC21B* pathogenic variants should be highlighted. The exact function of primary cilium in podocytes is unknown. There is evidence that primary cilium appears in fetal podocytes (undifferentiated cells), with the expression of *TTC21B* product (IFT139) in the cytoplasm and at the base of cilium (9). Primary cilium is lost after podocyte maturation (differentiated cells), but the expression of IFT139 persists in the microtubule network. This observation suggests that the glomerular damage in mature podocytes is not associated with cilium disruption, but with direct cytoskeleton dysregulation. This was also confirmed in differentiated podocytes with depleted IFT139 that led to actin cytoskeleton alteration, microtubule rearrangement, and cell size defect (9). The podocyte damage was rescued by the overexpression of wild type protein, in contrast to p.Pro209Leu protein, in which cytoskeleton damage remained. Based on this existing study, we can assume that further causative variants in *TTC21B* may also cause glomerular damage, and, thus, both heterozygous variants (also the novel p.Cys14Arg) found in our study contributed to disease development.

Our patient presented with brachydactyly and cone-shaped epiphyses. This bone anomaly has been reported in conorenal syndromes associated with chronic kidney disease. Retinitis pigmentosa and cerebral ataxia were also diagnosed in some affected patients (15). Huynh Cong et al. found *TTC21B* variants in seven families with tubuloglomerular disease (FSGS occurring along with tubulointerstitial damage). The age of presentation varied from childhood to early adulthood. High blood pressure was commonly present (9). Bullich et al. reported mutations in this gene in three families presenting with both glomerular (FSGS) and tubular disease. Hypertension and myopia presented as signs in some family members. The affected patients presented with non-nephrotic or nephrotic proteinuria resistant to immunosuppressive therapy and reached ESKD in childhood a few years after disease presentation (8). The authors also suspected the role of *TTC21B-*modifying alleles interacting with other disease-causing genes in glomerular and cystic diseases. This was also demonstrated by other researchers (7). Interestingly, *TTC21B* causative variants were also identified in patients with syndromic nephronophthisis associated with skeletal abnormalities. Two patients with Jeune Asphyxiating Thoracic Dystrophy phenotype have been described (7, 16).

## Conclusion

We present a unique case of genetic NS with tubulopathy and specific features, which contributes to the spectrum of *TTC21B-*associated disease. Our study underlies the importance of genetic examination for proper diagnostics and management of children with NS. Clinicians should suspect the mutated *TTC21B* gene in children with glomerular proteinuric disease with tubulointerstitial involvement, with or without skeletal abnormalities.

## Data Availability Statement

The original contributions presented in the study are included in the article/supplementary material, further inquiries can be directed to the corresponding author/s.

## Ethics Statement

All procedures performed in studies involving human participants were in accordance with the ethical standards of the Institutional Research Committee (Ethics Committee for Multi-Centric Clinical Trials of the Motol University Hospital and 2nd Faculty of Medicine, Charles University in Prague) and with the 1964 Helsinki declaration and its later amendments or comparable ethical standards. Written informed consent from the parents of the patient was obtained for the publication of the manuscript, any potentially identifiable images, or data included in this article.

## Author Contributions

MB and JZ performed the genetic analysis, participated in the patient's clinical care, and wrote the manuscript. SS, DZ, and JB participated in the patient's clinical care and collected the data. EH and MP helped with clinical data, examined the kidney biopsy, and critically reviewed the manuscript. All authors contributed to the article and gave final approval of the version to be published.

## Funding

This work was supported by the project (Ministry of Health, Czech Republic) for conceptual development of research organization 00064203 (University Hospital Motol, Prague, Czech Republic).

## Conflict of Interest

The authors declare that the research was conducted in the absence of any commercial or financial relationships that could be construed as a potential conflict of interest.

## Publisher's Note

All claims expressed in this article are solely those of the authors and do not necessarily represent those of their affiliated organizations, or those of the publisher, the editors and the reviewers. Any product that may be evaluated in this article, or claim that may be made by its manufacturer, is not guaranteed or endorsed by the publisher.
